# Investigating the effects of dexamethasone on blood-brain barrier permeability and inflammatory response following focused ultrasound and microbubble exposure

**DOI:** 10.7150/thno.40908

**Published:** 2020-01-01

**Authors:** Dallan McMahon, Wendy Oakden, Kullervo Hynynen

**Affiliations:** 1 Physical Science Platform, Sunnybrook Research Institute, Toronto, ON, Canada.; 2 Department of Medical Biophysics, University of Toronto, Toronto, ON, Canada.; 3 Institute of Biomaterials and Biomedical Engineering, University of Toronto, Toronto, ON, Canada.

**Keywords:** blood-brain barrier, dexamethasone, dynamic contrast-enhanced magnetic resonance imaging, inflammation, focused ultrasound

## Abstract

**Rationale**: Clinical trials are currently underway to test the safety and efficacy of delivering therapeutic agents across the blood-brain barrier (BBB) using focused ultrasound and microbubbles (FUS+MBs). While acoustic feedback control strategies have largely minimized the risk of overt tissue damage, transient induction of inflammatory processes have been observed following sonication in preclinical studies. The goal of this work was to explore the potential of post-sonication dexamethasone (DEX) administration as a means to mitigate treatment risk. Vascular permeability, inflammatory protein expression, blood vessel growth, and astrocyte activation were assessed.

**Methods**: A single-element focused transducer (transmit frequency = 580 kHz) and Definity^TM^ microbubbles were used to increase BBB permeability unilaterally in the dorsal hippocampi of adult male rats. Sonicating pressure was calibrated based on ultraharmonic emissions. Dynamic contrast-enhanced magnetic resonance imaging (DCE-MRI) was used to quantitatively assess BBB permeability at 15 min (baseline) and 2 hrs following sonication. DEX was administered following baseline imaging and at 24 hrs post-FUS+MB exposure. Expression of key inflammatory proteins were assessed at 2 days, and astrocyte activation and blood vessel growth were assessed at 10 days post-FUS+MB exposure.

**Results**: Compared to saline-treated control animals, DEX administration expedited the restoration of BBB integrity at 2 hrs, and significantly limited the production of key inflammation-related proteins at 2 days, following sonication. Indications of FUS+MB-induced astrocyte activation and vascular growth were diminished at 10 days in DEX-treated animals, compared to controls.

**Conclusions**: These results suggest that DEX provides a means of modulating the duration of BBB permeability enhancement and may reduce the risk of inflammation-induced tissue damage, increasing the safety profile of this drug-delivery strategy. This effect may be especially relevant in scenarios for which the goal of treatment is to restore or preserve neural function and multiple sonications are required.

## Introduction

The flexibility of using FUS+MB exposures to enhance the delivery of a variety of therapeutic agents to targeted brain regions has been demonstrated under numerous experimental conditions 1-6. By transiently increasing the permeability of the BBB 7, drugs can be delivered systemically and extravasate in the targeted locations to exert a therapeutic effect. Efficacious results in preclinical models of disease 8-10 have prompted the translation of this technique into human trials 11-18. While FUS+MB-mediated drug delivery may have the potential to aid in the treatment of several neuropathologies, the safety of modulating BBB permeability continues to spark debate 19-25, specifically in the context of diseases for which the aim of treatment is to preserve or improve long term neural function.

Broadly speaking, FUS+MB-mediated BBB permeability enhancement involves the intravenous administration of MBs, traditionally used as ultrasound contrast agents, and the propagation of ultrasound to spatially confined (I.e. focal volume), targeted locations within the brain. Mechanical stresses exerted on vascular walls by oscillating MBs drive subsequent changes in BBB permeability, thus controlling the *in vivo* behaviour of MBs is essential for producing predictable biological effects. To this end, strategies of calibrating the peak negative pressure (PNP) of sonication based on acoustic emissions - which can provide insight into the behaviour of MBs - have been developed 26,27 and continue to be refined 28-31. While the use of these acoustic feedback control strategies have largely minimized the risk of overt tissue damage (I.e. microhemorrhage, necrosis, substantial apoptosis), increased transcription of key inflammatory regulators (E.g. monocyte chemoattractant protein-1 (*Mcp1*) and intercellular adhesion molecule-1 (*Icam1*)) 22,32, blood vessel growth 33, and indications of astrocyte activation 32 have all been noted in preclinical studies following FUS+MB exposures that employ some form of acoustic feedback control. While there is debate regarding the degree, duration, and impact of these responses, it would be advantageous to develop strategies to mitigate the remaining risks.

DEX is a synthetic glucocorticoid used in a wide range of clinical applications, including the management of severe allergies, rheumatic diseases, and shock 34. Acting via glucocorticoid receptor binding, DEX can activate or suppress the transcription of specific genes controlled by glucocorticoid response elements. DEX can also act through non-transcriptional pathways, leading to the rapid activation of protein kinases 35. This can have broad reaching effects, including the activation of endothelial nitric oxide synthase, leading to altered blood flow and decreased vascular inflammation 35. In preclinical models, DEX has been shown to reduce inflammation and edema following intracerebral hemorrhage 36,37 and rapidly decrease vascular permeability in glioma 38,39. Clinically, DEX is commonly used in the management of glioblastoma multiforme-induced cerebral edema due to its low mineralocorticoid activity, long half-life, high potency, and ability to reduce BBB permeability 40. The effects of DEX on inflammation, cerebral edema, and BBB integrity, have also been employed in the context of mannitol-induced BBB permeability enhancement 41 as a response to adverse events in clinical trials 42.

The current study sought to determine if DEX administration following sonication alters vascular permeability, inflammation, blood vessel growth, and astrocyte activation. The overarching goal was to assess the ability of DEX to control the duration of increased BBB permeability and to mitigate risks of inflammation-induced tissue damage following FUS+MB exposure.

## Materials and methods

### Animals

Male Sprague Dawley rats (n = 40), weighing 230-330 g on the day of sonication, were used in this study (Taconic Biosciences, Germantown, NY, USA). Animals were housed in the *Sunnybrook Research Institute* animal facility (Toronto, ON, Canada) with access to food and water *ad libitum*. All animal procedures were approved by the *Animal Care Committee* at *Sunnybrook Research Institute* and are in accordance with the *Canadian Council on Animal Care* and *ARRIVE* guidelines.

### Study design

FUS+MB exposure was unilaterally targeted to the dorsal hippocampus, followed by quantitative MRI (I.e. T1-mapping and DCE-MRI) at 15 min post-sonication to assess BBB permeability. Saline or DEX (5 mg/kg; ip) was administered following imaging and animals were allowed to recover from anesthesia. At 2 hrs following sonication, quantitative MRI was repeated to determine the change in BBB permeability relative to 15 min post-FUS+MBs. A second dose of saline or DEX (5 mg/kg; ip) was administered 24 hrs following sonication in order to reduce potential inflammation related to extravasated bloodborne substances remaining from the period of elevated BBB permeability. For example, previous work has observed the presence of albumin in brain parenchyma 24 hrs following FUS+MB exposure 43, which may drive inflammatory processes 44. The supraphysiological dose of DEX administered in this study is at the high end of what has been employed clinically 45 and was based largely on preclinical research in rat models exploring the impact of DEX on brain vascular permeability 38,46-48.

**Figure 1:**
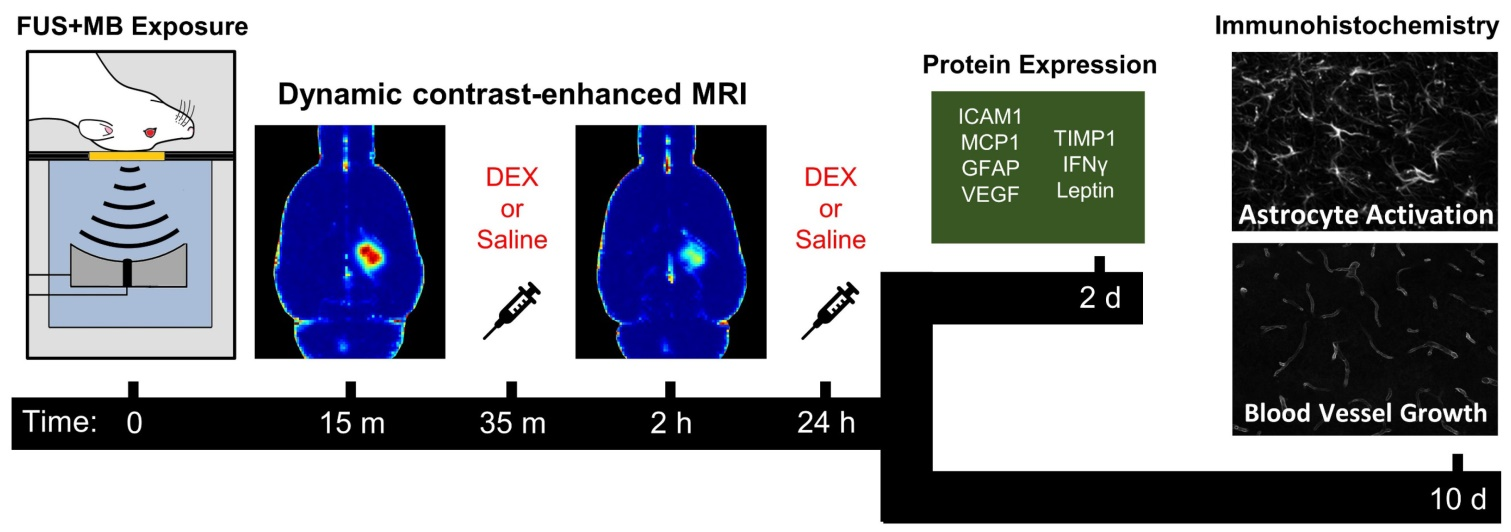
** Experiment Timeline.** FUS+MB exposure was unilaterally targeted to the dorsal hippocampus in each animal. Quantitative MRI (T1-mapping and DCE-MRI) was performed at 15 min following sonication to assess BBB permeability, afterwhich saline or DEX (5 mg/kg; ip) was administered. At 2 hrs following sonication, quantitative MRI was repeated to determine the change in BBB permeability relative to the 15 min time point. A second dose of saline or DEX (5 mg/kg; ip) was administered 24 hrs following sonication. Animals were sacrificed at either 2- or 10-days following sonication for protein expression and immunohistological analysis, respectively.

Prior to FUS+MB exposure, animals were randomized to receive either saline or DEX following sonication. Within these treatment groups, animals were further randomized to be sacrificed at either 2 days or 10 days post-FUS+MBs, for protein expression and immunohistological analysis, respectively. These time points were designed to capture changes in inflammatory protein expression, astrocyte activation, and vascular growth, based on previous work 19,32,33,49. The experiment timeline is graphically depicted in **Figure 1**.

### Animal preparation

Anesthesia was induced with 5% isoflurane and oxygen (1 L/min), then maintained at 1.5-2% isoflurane. During sonication and imaging, medical air was used as a carrier gas due to the impact of oxygen on MB circulation half-life 50,51. Hair overlaying the skull was removed with depilatory cream and a 22-gauge angiocath was placed in the tail vein. For the structural imaging and sonication, animals were secured in a supine position on an MRI-compatible sled, allowing transport between the bore of the MRI and the FUS system. The dorsal surface of the skull was coupled to a degassed, deionized water-filled polyimide window with ultrasound gel. Body temperature was maintained with heated saline bags. For quantitative MRI, animals were positioned prone to allow the receive coil to be placed in closer proximity to the brain. A bite bar and nose cone were used to secure the position of the head.

### FUS+MB exposure

**Figure 2:**
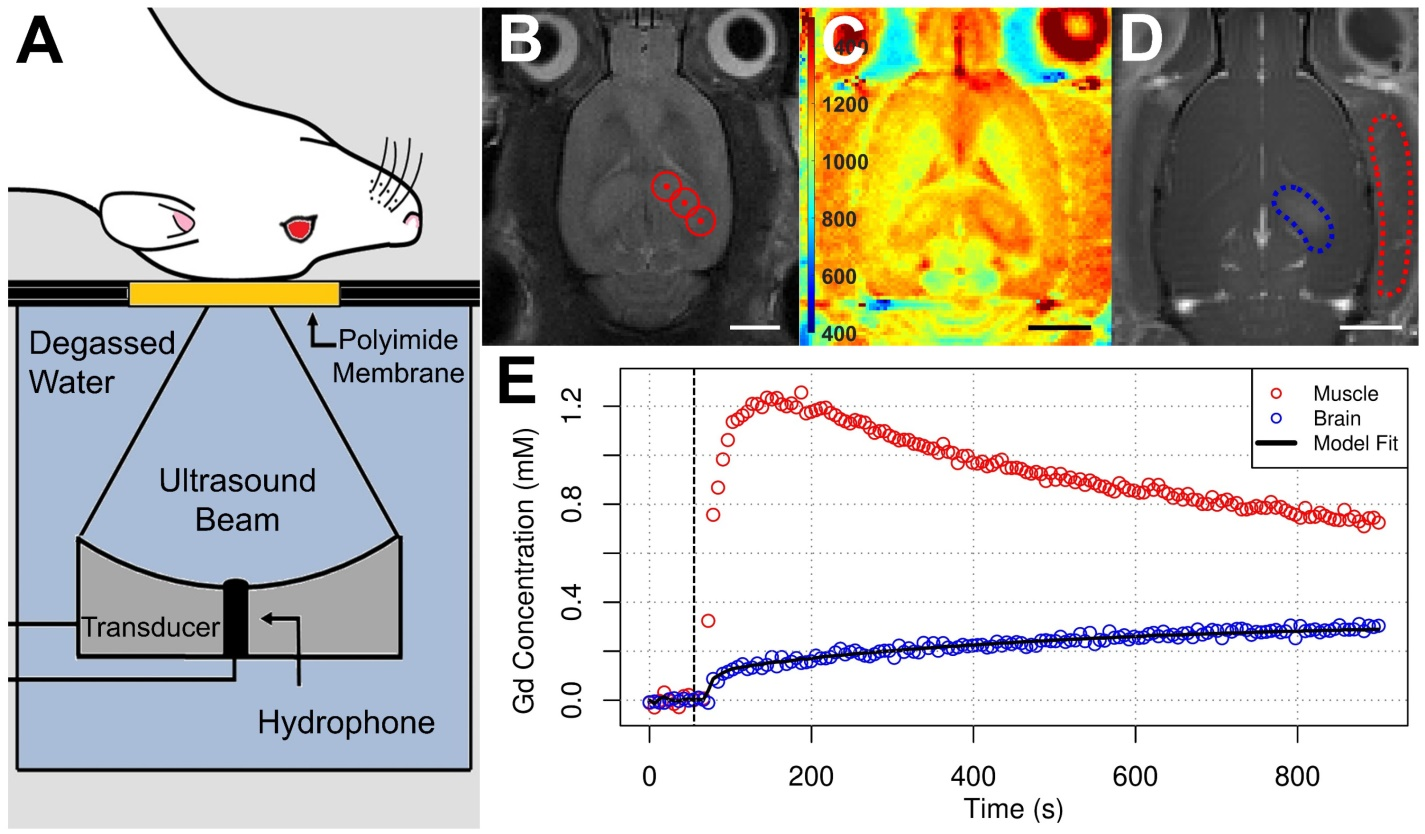
** MRI-Guided FUS+MB Exposure and Quantitative MRI.** (A) During structural imaging and sonication, rats were positioned supine on an MRI compatible sled with the dorsal surface of the head coupled to a polyimide membrane. The bottom of the membrane was coupled to a tank below filled with degassed, deionized water, housing the transducer/hydrophone assembly. (B) FUS was unilaterally targeted to the dorsal hippocampus based on T2w images. Quantitative MRI protocol consisted of (C) pre-contrast T1 mapping (colour bar indicates longitudinal relaxation time in ms) and (D) DCE-MRI (image depicts an average of the final 20 images captured). ROIs were drawn in the sonicated dorsal hippocampus and left temporal muscle based on pre-contrast inversion prepared RARE images (TI = 500 ms). (E) Contrast agent concentration was fit to a modified Tofts-Kermode model to estimate K^trans^ in the dorsal hippocampus. A reference-tissue (temporal muscle) method was used to estimate an arterial input function for this model. Scale bars = 4 mm.

Sonications were performed using an in-house developed prototype system (FUS Instruments Inc., Toronto, ON, Canada) equipped with a spherically focused transducer (focal number = 0.8, external diameter = 75 mm, transmit frequency (ƒ) = 580 kHz), calibrated using a planar fiber optic hydrophone with an active tip diameter of 10 μm (Precision Acoustics Ltd., Dorset, UK). The transducer was situated in a tank of degassed, deionized water and its movement was controlled with a motorized positioning system (3 degrees of freedom). Rapid motorized translation of the transducer enabled the sonication of several target locations in under one second (burst repetition frequency = 1 Hz). To allow ultrasound propagation from the transducer to the brain, the bottom of the polyimide membrane was coupled to the water tank below (**Figure 2a**). Coregistration of the transducer positioning system with MRI spatial coordinates allowed targets to be chosen from structural MR images. Three targets were placed in the left dorsal hippocampus of each animal (**Figure 2b**).

Ultrasound was delivered in 10 ms bursts with a burst repetition frequency of 1Hz for 120 sec. Acoustic emissions were monitored with an in-house manufactured PZT hydrophone located in a 25 mm opening in the centre of the transducer. To calibrate PNP, an acoustic control algorithm, similar to that described by O'Reilly and Hynynen 26, was employed. Briefly, starting PNP was set at 128 kPa (measured in water without skull attenuation) and increased by an increment of 8 kPa each second. MBs (20 μl/kg; Definity, Lantheus Medical Imaging, North Billerica, MA, USA), diluted in saline (1:9), were infused intravenously at a rate of 0.5 ml/min starting 10 sec into sonication. This delay was designed to allow baseline hydrophone measurements to be obtained without MBs in circulation. Once the magnitude of acoustic emissions at 1.5ƒ or 2.5ƒ passed the mean of baseline plus 10 standard deviations of the mean, the sonicating pressure was dropped by 50% and maintained at this level for the remainder of sonication. This strategy is designed to calibrate PNP based on *in vivo* MB response 26 and forms the basis of the acoustic feedback control algorithm currently employed for transcranial FUS+MB exposures in phase I clinical trials at *Sunnybrook Research Institute* 11,13-16.

### Retrospective acoustic emissions analysis

Hydrophone signals captured during each burst of the FUS+MB exposures (capture length = 11 ms, sampling rate = 20 MS/s) using a 14-bit scope card (ATS460; AlazarTech, Pointe-Claire, Quebec, Canada) were analysed retrospectively to explore potential relationships between BBB permeability enhancement (prior to DEX or saline administration) and spectral characteristics of the acquired acoustic emissions. At each target, the first 10 bursts were used as baseline measurements (MBs not in circulation). Fast Fourier transforms (FFT) were calculated for each burst to obtain signal spectra, from which specific frequencies of interest were analyzed (integration bandwidth = ± 0.2 kHz). The exposure-average magnitude of 0.5ƒ, ƒ, 1.5ƒ, 2ƒ, and wideband emissions were calculated for each target by subtracting the corresponding baseline signal values from each burst and then averaging across all bursts and targets within an animal. Wideband emissions were monitored at 890 kHz ± 5 kHz, corresponding to the peak sensitivity of the hydrophone. The peak magnitude of 0.5ƒ, ƒ, 1.5ƒ, 2ƒ, and wideband emissions were defined as the maximum signal value (after subtracting corresponding baseline signal values) at a single burst over the duration of sonication.

### MRI data acquisition

All MR experiments were conducted on a 7 T horizontal bore Avance BioSpec 70/30 scanner (Bruker BioSpin, Ettlingen, Germany) with a 20 cm inner diameter gradient insert coil with maximum gradient amplitude of 668 mT/m (Bruker BioSpin, Ettlingen, Germany). Images were acquired using an 8 cm inner diameter volume coil for transmit and a quadrature rat brain coil to receive (Bruker BioSpin, Ettlingen, Germany).

Structural T2w images used for FUS targeting were acquired using a RARE sequence with TE 46.2 ms, TR 4000 ms, and 1.0 mm slice thickness, prior to sonication. Targets were chosen in the sonication system software based on these images.

Quantitative MRI consisted of DCE-MRI with pre-contrast T1 mapping. A single slice with an axial orientation at the level of the dorsal hippocampus was selected for imaging. Identical slice location and geometry were used for all quantitative MRI. For the DCE-MRI, a FLASH sequence with TE 2.175 ms, TR 20 ms, 20° flip angle, 3 averages, matrix size 100 x 100, field of view 24 mm x 24 mm, and slice thickness 1.0 mm, was acquired at a temporal resolution of 6.0 sec for 15 min. A bolus of gadobutrol (0.4 mmol/kg; Gadovist, Bayer AG, Leverkusen, Germany) was administered intravenously after 1 min (10 pre-contrast images), followed by an additional 14 min of imaging.

T1 mapping was performed immediately prior to DCE-MRI using an inversion recovery RARE sequence with TE 7 ms, TR 5000 ms, rare factor of 16, 1 average, matrix size 100 x 100, field of view 24 x 24 mm^2^, slice thickness 1.0 mm, and 5 inversion times: 125, 250, 500, 1500, and 4500 ms.

### DCE-MRI analysis

To quantitatively assess BBB permeability, the transfer constant (K^trans^) of gadobutrol from plasma to extravascular-extracellular space (EES) was calculated from T1-mapping and DCE-MRI. K^trans^ measurements were obtained at 15 min and 2 hrs post-FUS+MBs, denoted as K^trans, 15 min^ and K^trans, 2 hrs^, respectively. The relationship between the concentration of contrast agent and change in relaxation rate can be expressed as:


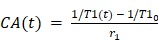
 (1)

where CA(t) is contrast agent concentration as a function of time, r_1_ is the longitudinal relaxivity of gadobutrol (4.2 s^-1^ mM^-1^ in human whole blood at 37^o^C in a 7T field 52), T1(t) is the T1 of tissue as a function of time, and T1_0_ is the T1 of tissue in the absence of contrast agent.

T1_0_ within each region of interest or on a voxel-by-voxel basis (**Figure 2c**) was calculated from the inversion recovery RARE images acquired immediately prior to DCE-MRI. The inversion recovery data was fit to the following equation using Matlab (Mathworks Inc., Natick, MA, USA):


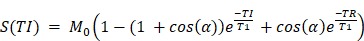
 (2)

where the initial magnetization (M_0_), the error in flip angle (α) and T1 are the free parameters. Contrast agent concentration was fit to a modified Tofts-Kermode model 53 that accounts for the presence of separate intravascular and extravascular extracellular compartments (**Figure 2E**). Least squares regression was used for fitting. The tissue concentration of gadobutrol was modeled with the following equation:


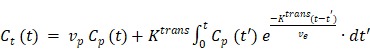
 (3)

where C_t_(t) is the concentration of contrast agent in tissue (calculated using Equation 1) as a function of time, K^trans^ is the transfer rate constant from the intravascular space to the EES, v_p_ and v_e_ are the plasma volume and distribution volume of contrast agent in the EES (per unit volume of tissue), respectively. C_p_(t), the plasma concentration of gadobutrol as a function of time (arterial input function, AIF), was estimated using a reference-tissue method 54,55.

The time-dependent concentration of gadobutrol in temporal muscle (C_muscle_; calculated using Equation 1) was used to derive AIF using literature values of K^trans, muscle^ (0.11 min^-1^) and v_e, muscle_ (0.20) in rat muscle 54 as follows:


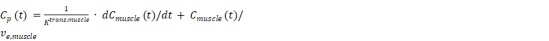
 (4)

This data-driven AIF approach has previously been shown to produce accurate estimates of K^trans^ when compared to direct measurement 56,57 and population-derived AIF approaches 55. For estimates of K^trans^ in the dorsal hippocampus, a region of interest was drawn based on pre-contrast inversion prepared RARE images (TI = 500 ms).

### Protein analysis

Two days following sonication, animals that were randomized to be included in protein analyses were transcardially perfused with ice-cold phosphate buffer (PB; 0.1M, pH 7.4). Dorsal hippocampi were rapidly dissected on ice, frozen with dry ice, and stored at -80 °C until further processing. For protein extraction, tissue was placed in 1x RIPA buffer with protease inhibitors (ab65621, Abcam Inc, Cambridge, MA, USA) on ice at a concentration of 10 μl/mg of tissue. Samples were homogenized via sonication (Sonifier 250, Branson Ultrasonics, Danbury, CT, USA) then centrifuged at 15000 g for 20 min at 4 °C. Supernatant was aliquoted and stored at -80 °C until analysis. BCA assay (Thermo Scientific, Waltham, MA, USA) was used to determine total protein concentration for each sample.

Rat Cytokine Array Q2 (Raybiotech, Norcross, GA, USA) was used to assess concentrations of MCP1, ICAM1, interferon gamma (IFNɣ), interleukin-10 (IL10), IL1β, IL6, leptin (LEP), L-selectin, tissue inhibitor matrix metalloproteinase-1 (TIMP1), and tumour necrosis factor alpha (TNFɑ). The assay was performed as per manufacturer's instructions using a total protein concentration of 1000 μg/ml. To assess the concentrations of GFAP and vascular endothelial growth factor (VEGF), enzyme-linked immunosorbent assays (ELISAs) were performed in accordance with manufacturer's instructions (GFAP: ab233621, Abcam Inc, Cambridge, MA, USA; VEGF: ab100787, Abcam Inc, Cambridge, MA, USA). Total protein concentrations of 1 μg/ml and 100 μg/ml were used for GFAP and VEGF ELISAs, respectively. All protein concentrations are expressed as a ratio of sonicated to non-sonicated hippocampi within each animal.

### Immunohistochemistry

Ten days following sonication, animals that were randomized to be included in immunohistochemical analyses were transcardially perfused with ice-cold PB (0.1M, pH 7.4), followed by 4% paraformaldehyde in PB. Brains were extracted, post-fixed for 24 hrs at 4 °C, then transferred to 30% sucrose in PB and stored at 4 °C until fully saturated (~3 days). Brains were embedded in optimum cutting temperature compound (Tissue-Tek, Torrance, CA, USA) and stored at -80 °C until cryostat sectioning. Coronal sections (35 μm thick) were stored in cryoprotectant (glycerin, ethylene glycol, and 0.2M PB in a ratio of 2:3:5, respectively) at -10°C until immunohistological processing.

Free-floating sections were blocked for 1 hr at room temperature (0.1% Triton X-100, 1% bovine serum albumin, 2% goat serum, 1x PBS), then incubated in rabbit anti-GFAP primary antibodies (1:800; ab7260, Abcam Inc, Cambridge, MA, USA) for 48 hrs at 4 °C. Sections were incubated in goat anti-rabbit IgG Alexa Fluor 647 secondary antibody (1:800; ab150079, Abcam Inc, Cambridge, MA, USA) for 24 hrs at 4 °C, then mounted onto charged glass slides (X-tra, Leica Microsystems, Wetzlar, Germany) and coverslipped with aqueous mounting media (Fluoroshield™ with DAPI, Sigma-Aldrich Corporation, St. Louis, MO, USA). Three 10-min washes were performed following each step, except blocking. Slides were stored in the dark at 4 °C until imaging.

Staining for blood vessels followed the same protocol as above with the addition of an antigen retrieval step. Prior to primary antibody incubation, sections were placed in 2 M HCl for 90 min, followed by 10 min in 0.1 M borate buffer (pH 8.5). A combination of rabbit anti-cluster of differentiation-31 (CD31; 1:800; ab28364, Abcam Inc, Cambridge, MA, USA) and rabbit anti-glucose transporter-1 (GLUT1; 1:800; ab15309, Abcam Inc, Cambridge, MA, USA) primary antibodies were used to produce more complete blood vessel labeling.

### Confocal imaging

For quantification of GFAP immunoreactive density and blood vessel density by size, image stacks (3 μm spacing, 0.60 μm/pixel, 1024×1024-pixel field of view) were collected through the entire thickness of each section, bilaterally, in each of three hippocampal subfields, CA1, CA4, and the DG. Sections (7-9 per animal) were imaged with a 20x objective (NA 0.75) using a confocal laser scanning microscope system (A1+, Nikon, Tokyo, Japan) at excitation wavelengths of 639.1 nm and 403.1 nm for GFAP/GLUT1/CD31 and DAPI, respectively. Emissions were received at 663-738 nm and 425-475 nm, respectively. All imaging parameters (E.g. laser power, gain, scan-speed, etc.) were kept constant across sections and animals to allow for more accurate quantitative analyses. To ensure subsequent image analysis was performed on consistent regions of the dorsal hippocampus, image stacks were cropped to include only stratum radiatum of CA1, between the blades of the DG for CA4, and the molecular layer of the DG. Image stacks were also trimmed in the z-direction, keeping 5 images starting at the first complete optical section.

### Confocal image analysis

GFAP positive astrocytes were segmented using an in-house designed ImageJ pipeline. The *Tubeness* plugin (ImageJ), with sigma values of 0.2, 0.4, and 0.75, was used to detect *tube-like* structures within each image. This plugin uses eigenvalues of the Hessian matrix to calculate a measure of *tubeness* for each pixel within an image 58. Using larger values of sigma, thicker tubes are detected. The sigma values used in this study were empirically chosen to highlight the full range of sizes of astrocytic processes. Once detected, structures were segmented by auto-thresholding and binary masking. GFAP immunoreactive density for each image stack was calculated as a sum of densities from all images, then averaged across all image stacks in each hemisphere. Density measurements are expressed as a ratio of sonicated to non-sonicated dorsal hippocampi for each animal.

Maximum intensity projections of GLUT1/CD31 stained sections were used to quantify the density of blood vessel segments by size. Using the *ObjectJ* plugin (ImageJ), the diameter of each blood vessel segment was manually measured in each image by an author (DM) blinded to treatment. For each animal, a histogram of blood vessel segment frequency, binned to diameters of <5 μm, 5-7.5 μm, 7.5-10 μm, and >10 μm, was normalized to imaging volume to calculate density. Mean densities of blood vessel segments for each bin are expressed as a relative difference between sonicated and non-sonicated dorsal hippocampus for each animal.

### Statistics

All statistical analyses were performed using R 3.4.3. For the comparison of dorsal hippocampal K^trans, 15 min^ to K^trans, 2 hrs^, between saline and DEX-treated animals, analysis of covariance (ANCOVA) was used to assess statistical significance. Differences in the density of blood vessel segments and protein expression between sonicated and non-sonicated hemispheres within experimental groups were assessed by repeated measures one-way analysis of variance and post-hoc paired student's t-tests (two-tailed). False discovery rate correction for multiple comparisons was used to account for the simultaneous measurement of 5 proteins by rat cytokine array and for the assessment of 4 bins of blood vessel diameters. Differences in GFAP immunoreactive density between sonicated and non-sonicated hemispheres within experimental groups were assessed by paired student's t-tests (two-tailed). Linear least-squares regression was used to assess the relationship between dorsal hippocampal K^trans, 15 min^ and both the peak and exposure-average magnitude of acoustic emissions at specific frequencies. For all analyses, a p-value of 0.05 was used as the threshold for statistical significance. Unless otherwise specified, variance is expressed as standard deviation of the mean.

## Results

### BBB permeability enhancement following sonication

BBB permeability was assessed at 15 min and 2 hrs post-FUS+MBs by quantitative MRI. K^trans, 15 min^ in the sonicated dorsal hippocampus, prior to the administration of DEX or saline, ranged from 0.0023 min^-1^ to 0.0231 min^-1^. The K^trans, 15 min^ values reported here are consistent with previous observations following FUS+MB exposure 59-62.

For inclusion in all analyses focused on the effects of DEX, a minimum K^trans, 15 min^ threshold was set at 0.005 min^-^. This threshold was designed to ensure that changes in BBB permeability were detectable 2 hrs following sonication, enabling an accurate assessment of the changes in vascular permeability relative to 15 min post-FUS+MBs. Of the 40 animals that underwent unilateral dorsal hippocampal FUS+MB exposure and DCE-MRI, 29 satisfied this criterion and 11 were excluded. Of these 29 animals, 14 were treated with saline and 15 were treated with DEX following sonication.

Prior to saline or DEX administration, no significant difference was detected in dorsal hippocampal K^trans, 15 min^ between groups (saline = 0.0132 min^-1^ ± 0.0059 min^-1^; DEX = 0.0128 min^-1^ ± 0.0047 min^-1^; p = 0.85). Similarly, no significant differences were detected in dorsal hippocampal K^trans, 15 min^ between groups sacrificed at 2 days (saline = 0.0136 min^-1^ ± 0.0050 min^-1^; DEX = 0.0138 min^-1^ ± 0.0045 min^-1^; p = 0.95) or 10 days (saline = 0.0128 min^-1^ ± 0.0070 min^-1^; DEX = 0.0121 min^-1^ ± 0.0050 min^-1^; p = 0.80) following sonication. No significant difference was detected in contralateral dorsal hippocampal K^trans, 15 min^ between groups (saline = 5.31 x 10^-6^ min^-1^ ± 3.17 x 10^-6^ min^-1^; DEX = 6.42 x 10^-6^ min^-1^ ± 4.81 x 10^-6^ min^-1^; p = 0.47; **Supplementary Figure 1**).

### Correlations between acoustic emissions and BBB permeability

During sonication, PNP was increased until acoustic emissions at 1.5ƒ or 2.5ƒ were detected above baseline. Maximum PNP values (triggering PNP) were 362 kPa ± 74 kPa and 388 kPa ± 72 kPa, in DEX and saline-treated animals, respectively (p = 0.27). PNP was maintained at 50% of the triggering pressure for the remainder of sonication.

Due to software errors, complete scope card data were captured for only 32 of 40 animals. The 8 animals with truncated data were excluded from retrospective acoustic emissions analysis; however, no minimum K^trans, 15 min^ threshold was set for inclusion.

**Figure 3:**
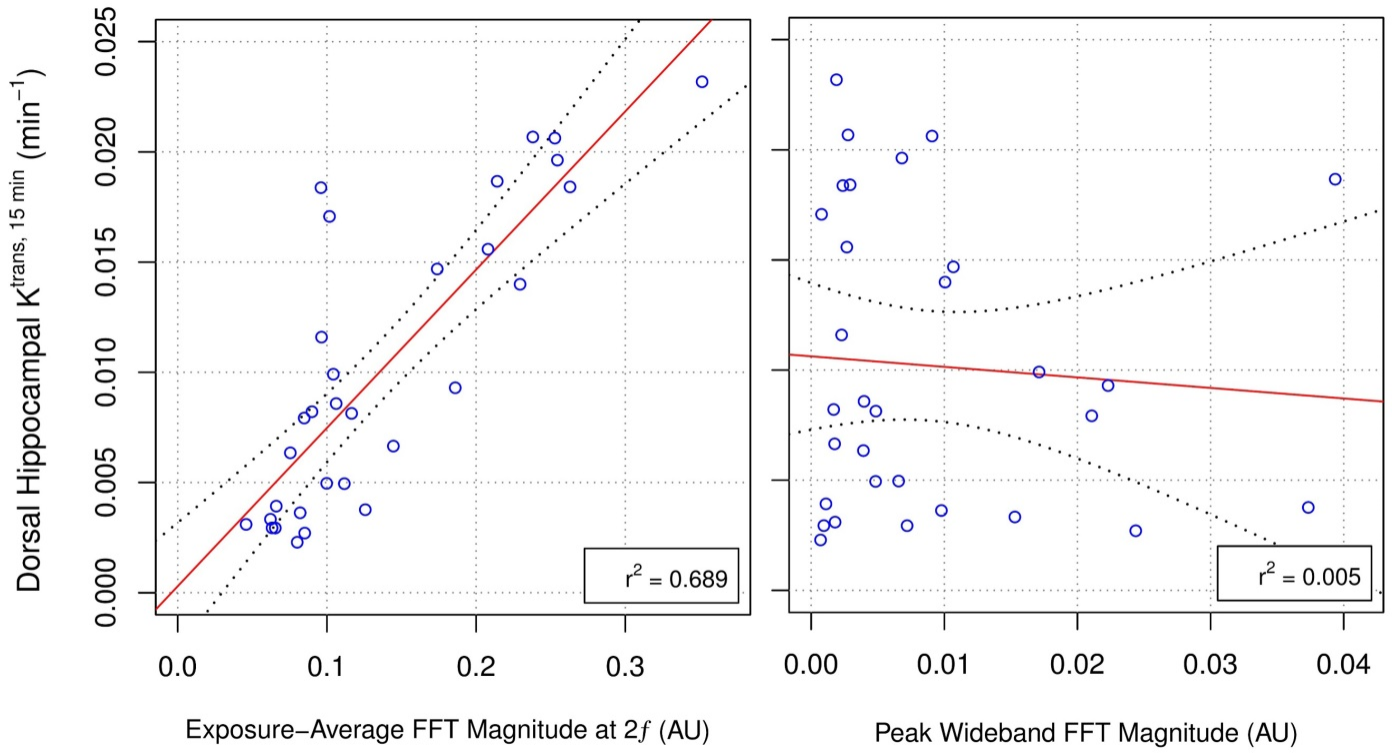
** Correlations Between Acoustic Emissions and Dorsal Hippocampal K^trans, 15 min^.** Hydrophone signals captured during FUS+MB exposures were analysed retrospectively to explore potential relationships between K^trans, 15 min^ measurements (prior to saline or DEX administration) and spectral characteristics of the acquired acoustic emissions. The exposure-average magnitude of 2ƒ emissions displayed a strong linear correlation to K^trans, 15 min^ in the sonicated dorsal hippocampus (r^2^ = 0.689). Peak wideband emissions did not explain a significant portion of the variance in K^trans, 15 min^ measurements. These results suggest that the changes in BBB permeability observed in this study were not driven inertial cavitation. Black dotted lines indicate 95% confidence intervals. AU = arbitrary units.

Retrospective analysis of the exposure-average magnitude of 2ƒ emissions showed a strong linear correlation to dorsal hippocampal K^trans, 15 min^ (r^2^ = 0.689; **Figure 3**). The exposure-average magnitude of 0.5ƒ, ƒ, 1.5ƒ, or wideband emissions showed no strong correlations to dorsal hippocampal K^trans, 15 min^, with r^2^ values of 0.012, 0.156, 0.107, and 0.004, respectively. Similarly, linear regression analysis of the peak magnitude of 0.5ƒ, ƒ, 1.5ƒ, 2ƒ, or wideband emissions during sonication versus K^trans, 15 min^ displayed r^2^ values of 0.033, 0.046, 0.0264 0.597, and 0.005, respectively (**Supplementary Figure 2**).

### Effects of DEX on BBB permeability

**Figure 4:**
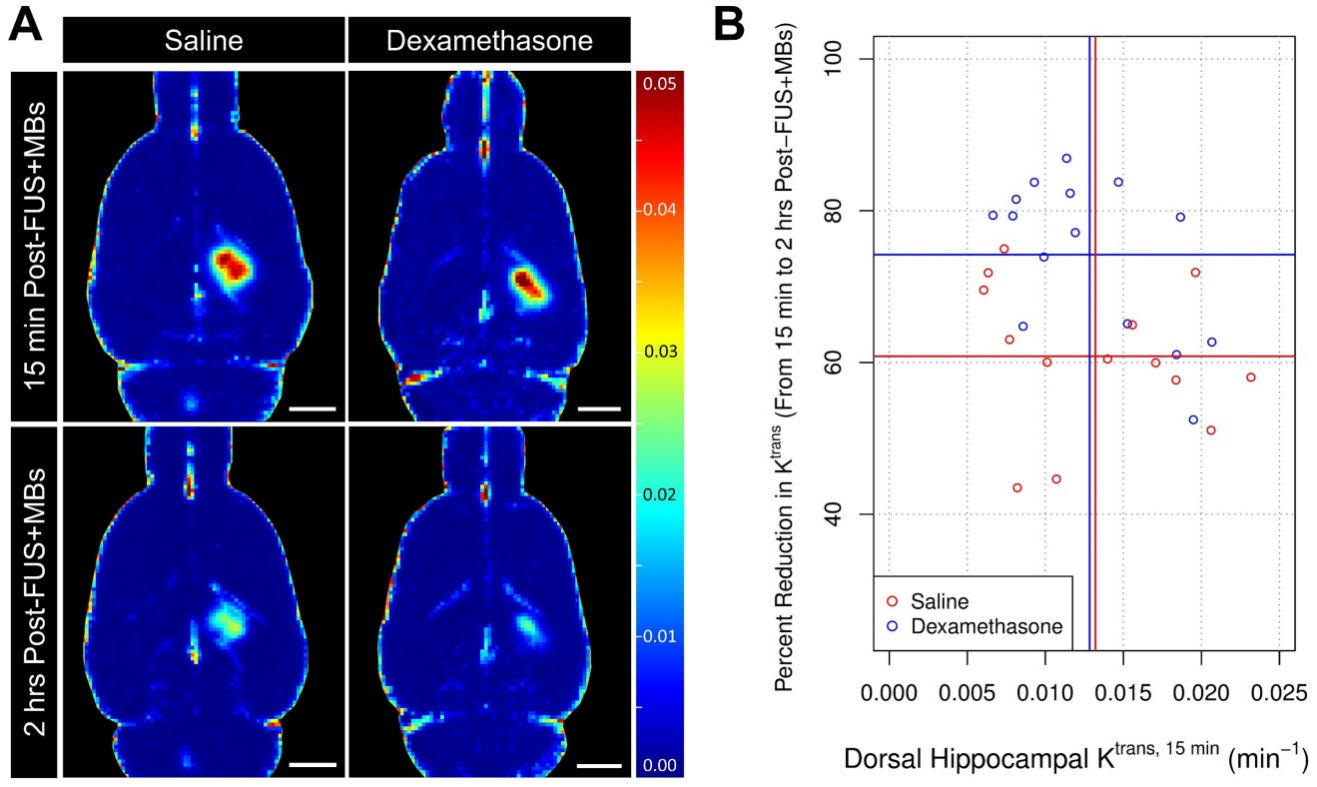
** Impact of DEX on BBB Permeability Enhancement Following FUS+MB Exposure.** (A) Representative K^trans^ maps acquired at 15 min and 2 hrs post-FUS+MB exposure demonstrate a more rapid restoration of BBB integrity in a DEX-treated animal compared to a saline-control animal. (B) DEX administration resulted in a significantly greater reduction in mean dorsal hippocampal K^trans^ from 15 min to 2 hrs post-FUS+MBs (74.2% ± 10.4%), compared to saline administration (60.8% ± 9.7%). p = 0.003 (ANCOVA). No significant differences were detected in mean dorsal hippocampal K^trans, 15 min^ between saline and DEX-treated animals prior to administration. Vertical and horizontal lines represent group means relative to their respective axes. n = 14 saline-treated and 15 DEX-treated animals. Scale bars = 3 mm.

To determine its impact on BBB permeability following FUS+MBs, DEX was administered immediately following K^trans, 15 min^ measurements. ANCOVA was used to assess the effect of treatment (saline vs DEX) on the change in dorsal hippocampal K^trans^ between 15 min and 2 hrs following sonication. Compared to saline-control (n = 14), DEX (n = 15) was found to significantly reduce K^trans, 2 hrs^ after administration (p = 0.003). At 2 hrs following sonication, K^trans^ had dropped by 60.8% ± 9.7% and 74.2% ± 10.4% in animals that received saline and DEX, respectively (**Figure 4b**). This result suggests that DEX significantly alters BBB permeability following FUS+MBs, acting to expedite the restoration of BBB integrity.

### Expression of inflammatory markers

**Figure 5:**
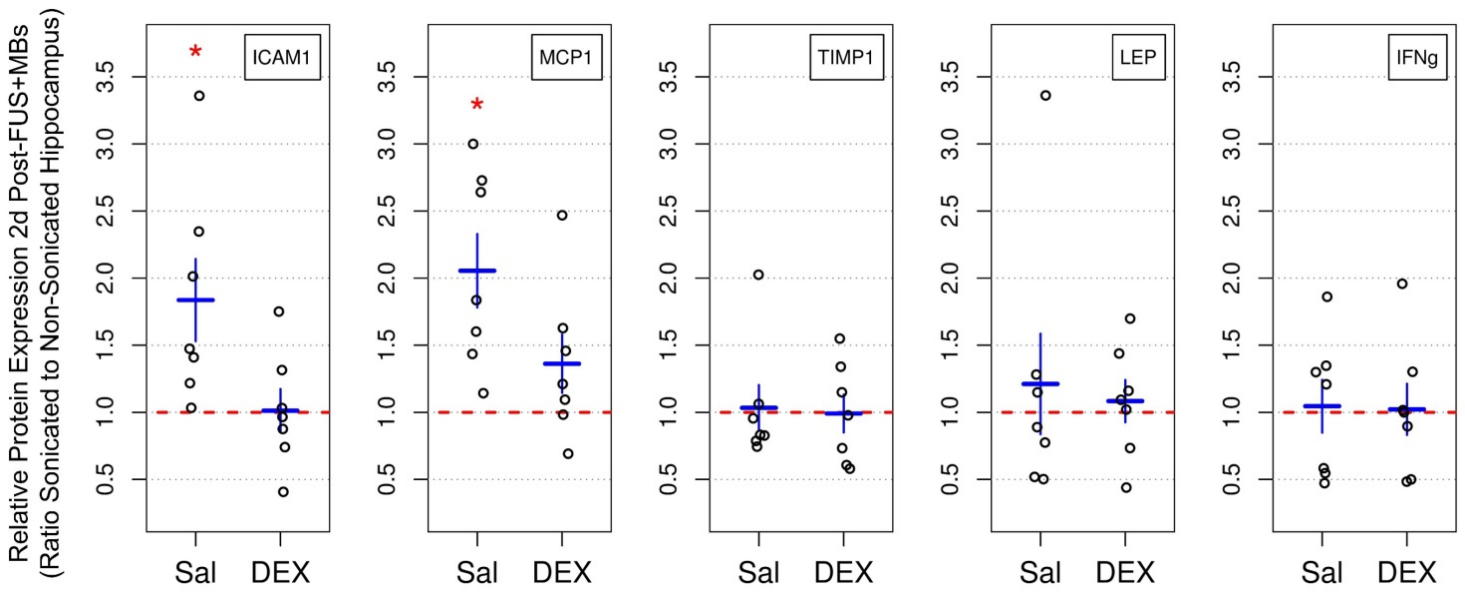
** Relative Protein Expression 2 Days Post-FUS+MB Exposure.** The expression of select inflammatory markers were assessed bilaterally in dorsal hippocampi 2 days following sonication by rat cytokine array. The mean ratios of ICAM1 and MCP1 expression in the sonicated to non-sonicated dorsal hippocampi for saline-treated animals was 1.83 ± 0.81 (p = 0.049) and 2.05 ± 0.72 (p = 0.049), respectively. No significant differences in the expression of TIMP1, LEP, and IFNg were detected between hemispheres. Animals receiving DEX following FUS+MBs (n = 7) did not display significant differences between sonicated and non-sonicated dorsal hippocampi in the expression of any of the proteins assessed. * indicates p < 0.05, paired student's t-test, corrected for multiple comparisons. Red, horizontal, dashed line indicates no difference between sonicated and non-sonicated dorsal hippocampi. Error bars represent standard error of the mean. n = 7 saline-treated and 7 DEX-treated animals.

The expression of key inflammatory markers were assessed bilaterally in the dorsal hippocampi 2 days following sonication by rat cytokine array (**Figure 5**). The mean ratios of ICAM1 and MCP1 expression in the sonicated to non-sonicated dorsal hippocampi for saline-treated animals (n = 7) were 1.83 ± 0.81 (p = 0.049) and 2.05 ± 0.72 (p = 0.049), respectively. No significant differences in the expression of TIMP1, LEP, and IFNg were detected between hemispheres. No significant differences between sonicated and non-sonicated dorsal hippocampi in the expression of any of proteins assessed were observed in DEX-treated animals (n = 7). For proteins of interest with more than 25% of samples below the dynamic range of measurement (IL10, IL1β, IL6, SELL, and TNFɑ), analysis of differential expression between hemispheres was not performed.

### GFAP expression and immunoreactivity

**Figure 6:**
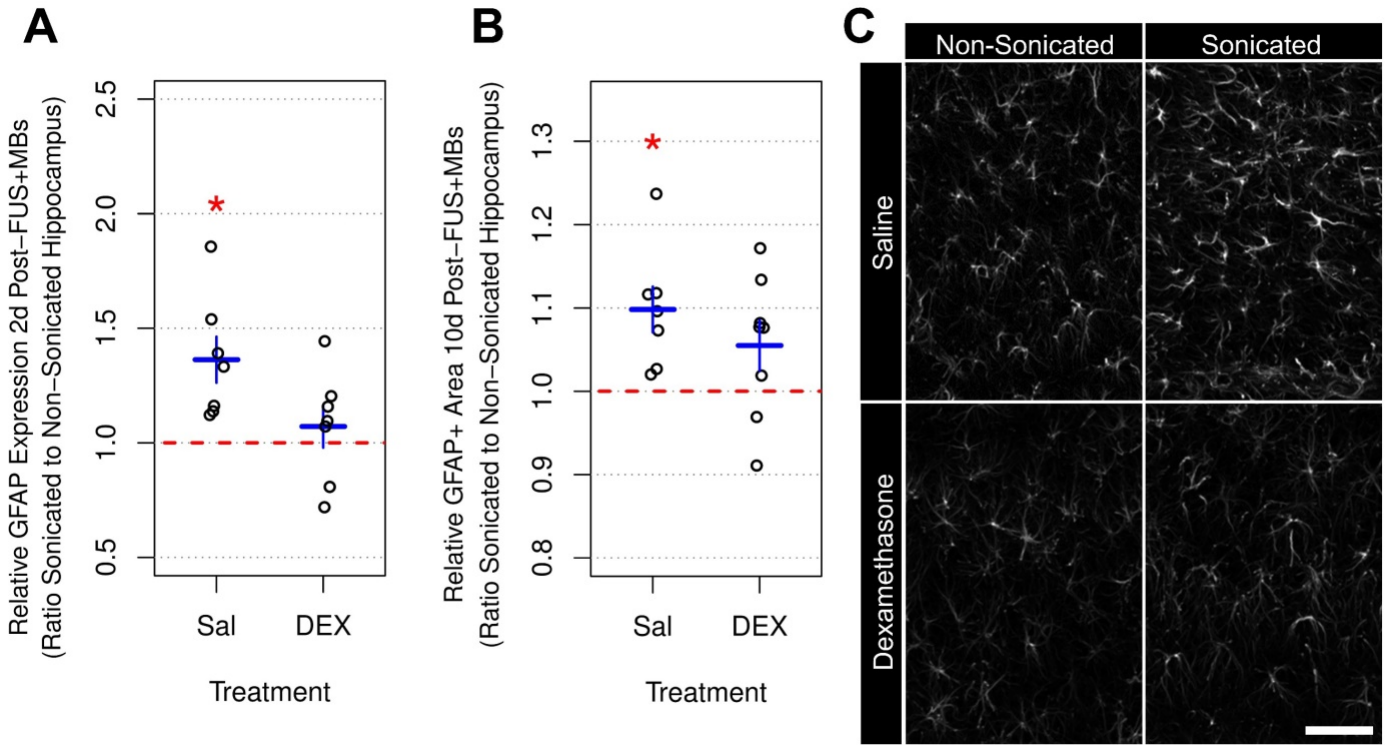
** Relative GFAP Expression and Immunoreactive Density Following FUS+MB Exposure.** (A) Two days following sonication, the mean ratio of GFAP expression in the sonicated to non-sonicated dorsal hippocampi for saline-treated animals was 1.36 ± 0.26 (p = 0.005). Animals receiving DEX displayed no significant differences (p = 0.56). (B) Ten days post-FUS+MBs, the mean ratio of GFAP immunoreactive density in the sonicated to non-sonicated dorsal hippocampi for saline-treated animals was 1.10 ± 0.07 (p = 0.01). Animals receiving DEX did not display a significant difference in dorsal hippocampal GFAP immunoreactive density between hemispheres (mean ratio of 1.06 ± 0.09 for GFAP immunoreactive density in the sonicated to the non-sonicated dorsal hippocampi; p = 0.11). (C) Representative images of GFAP immunoreactivity 10 days following FUS+MB exposure demonstrating reactive astrocytes in the sonicated hippocampus of saline-treated animals. * indicates p < 0.05, paired student's t-test. Red, horizontal, dashed line indicates no difference between sonicated and non-sonicated dorsal hippocampi. Error bars represent standard error of the mean. n = 7 saline-treated and 7 DEX-treated animals for analysis 2 days post-FUS+MB exposure. n = 7 saline-treated and 8 DEX-treated animals for analysis 10 days post-FUS+MB exposure. Scale bar = 100 µm.

Elevations in GFAP protein expression 49 and immunoreactivity 19,25,49 have previously been observed following FUS+MB-mediated BBB permeability enhancement. In the current study, dorsal hippocampi were bilaterally dissected 2 days following sonication and GFAP expression was measured by ELISA (**Figure 6a**). The mean ratio of GFAP expression in the sonicated to non-sonicated dorsal hippocampi for saline-treated animals (n = 7) was 1.36 ± 0.26 (p = 0.005). Conversely, animals receiving DEX (n = 7) displayed no significant differences between hemispheres; the mean ratio of GFAP expression in the sonicated to non-sonicated dorsal hippocampi for DEX-treated animals was 1.07 ± 0.24 (p = 0.56).

GFAP immunoreactive density was assessed 10 days post-FUS+MB exposure in tissue sections using confocal imaging and an automated image analysis pipeline (**Figure 6b**). The mean ratio of GFAP immunoreactive density in the sonicated to non-sonicated dorsal hippocampi for saline-treated animals (n = 7) was 1.10 ± 0.07 (p = 0.01). Animals receiving DEX (n = 8) did not display a significant difference in dorsal hippocampal GFAP immunoreactive density between hemispheres, with a mean ratio of 1.06 ± 0.09 (p = 0.11) for the sonicated to the non-sonicated hemispheres; however, the current study may be underpowered to detect significant differences in GFAP immunoreactive density, given the larger variance and smaller effect size in DEX-treated animals compared to saline-controls.

### Vascular changes

**Figure 7:**
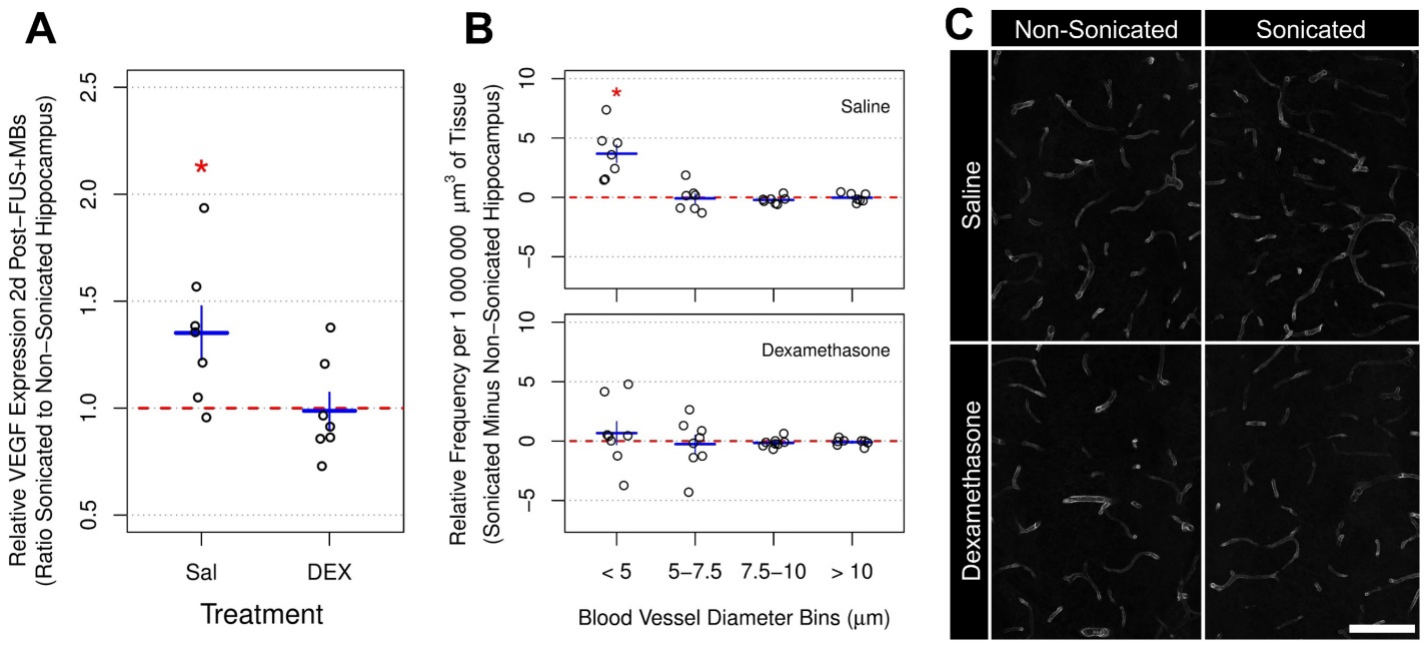
** Relative VEGF Expression and Vascular Density Following FUS+MB Exposure.** (A) Two days post-FUS+MBs, the mean ratio of VEGF expression in the sonicated to non-sonicated dorsal hippocampi for saline-treated animals was 1.35 ± 0.33 (p = 0.025). This effect was not present in animals receiving DEX following FUS+MBs (p = 0.83). (B) The density of small blood vessel segments (diameter < 5 μm) was significantly greater in the sonicated hippocampus of saline-treated animals compared to the non-sonicated hippocampus (3.67 ± 2.11 more small blood vessel segments per 1 000 000 μm^3^ than non-sonicated hippocampus; p = 0.015). No significant differences were observed for other sizes of blood vessels. Animals treated with DEX displayed no significant differences in blood vessel frequency between hemispheres for any size of vasculature. (C) Representative images of hippocampal vasculature 10 days following FUS+MB exposure demonstrating a small increase the density of small blood vessels in the sonicated hippocampus of saline-treated animals. * indicates p < 0.05, paired student's t-test. Red, horizontal, dashed line indicates no difference between sonicated and non-sonicated dorsal hippocampi. Error bars represent standard error of the mean. n = 7 saline-treated and 7 DEX-treated animals for analysis 2 days post-FUS+MB exposure. n = 7 saline-treated and 8 DEX-treated animals for analysis 10 days post-FUS+MB exposure. Scale bar = 100 µm.

VEGF has well-established roles in vascular growth and remodeling. Previous work has demonstrated changes in VEGF expression following FUS+MB exposure 19,33. In the current study, 2 days post-FUS+MB exposure, the mean ratio of VEGF expression in the sonicated to non-sonicated dorsal hippocampi (**Figure 7a**) for saline-treated animals was 1.35 ± 0.33 (p = 0.025). However, as with GFAP, ICAM1, and MCP1 expression, animals receiving DEX (n = 7) displayed no significant differences between hemispheres; the mean ratio of VEGF expression in the sonicated to non-sonicated dorsal hippocampi for DEX-treated animals was 1.06 ± 0.22 (p = 0.83).

To assess blood vessel growth, the frequency of blood vessel segments per unit volume of brain tissue was compared across the dorsal hippocampi between sonicated and non-sonicated hemispheres 10 days post-FUS+MBs (**Figure 7b**). For this analysis, the diameters of blood vessel segments were measured and binned by size. Consistent with a previous report 33, the volume-adjusted frequency of small blood vessel segments (diameter < 5 μm) was significantly greater in the sonicated hippocampus of saline-treated animals (n = 7) compared to the non-sonicated hippocampus (3.67 ± 2.11 more small blood vessel segments per 1 000 000 μm^3^ than non-sonicated hippocampus; p = 0.015). No significant differences were observed for other sizes of blood vessels. Animals treated with DEX (n = 8) displayed no significant differences in blood vessel frequency between hemispheres for any size of vasculature.

## Discussion

The results presented here explore the effects of post-sonication DEX administration on vascular permeability, inflammation, blood vessel growth, and astrocyte activation. While previous work has demonstrated that overt tissue damage can be largely minimized with the use of acoustic feedback control 26,28, transient effects on tissue health have been noted under experimental conditions that calibrate PNP based on MB activity 22,29,32,33,63-65. Given their magnitude and duration, the long-term impacts of these changes are unlikely to represent a prohibitive risk; however, multiple FUS+MB exposures with a high repetition frequency (E.g. weekly) may result in the accumulation of detrimental effects. Additionally, as with any medical intervention, there is a non-zero risk of adverse events (E.g. microhemorrhages, infection, edema). DEX administration may help to address these safety concerns by providing a means to expedite the restoration of BBB integrity and to reduce inflammation following FUS+MB exposure.

In the present study, post-sonication DEX administration was found to alter the dynamics of vascular permeability in healthy brain tissue, leading to a significantly greater reduction in K^trans^ relative to saline-controls at 2 hrs following FUS+MB exposure. This rapid effect on BBB permeability has previously been characterized in the context of C_6_ glioma, with Shapiro *et al.* noting a 37% reduction in the K^trans^ of ^14^C-alpha aminoisobutyric acid 1 hr following injection of DEX (10 mg/kg; ip), relative to baseline measurements 38. Similarly, significant reductions in the K^trans^ of gadopentetate dimeglumine in glioblastoma vasculature have been reported 48-72 hrs following DEX administration (16 mg/day) in human patients 66.

Mechanisms through which DEX may act to alter BBB permeability include: increasing the expression of occludin 48,67 and zonula occludens-1 67, preventing TNFɑ-dependent trafficking of tight junction proteins 68, altering vascular tone 69 and mean arterial pressure 47, and reducing cytokine-induced expression of matrix metalloproteinase-9 70. Given the rapidity of the effects observed in this study, it could be hypothesized that non-transcriptional actions of DEX on mean arterial pressure and/or TJ protein trafficking play a role in reducing BBB permeability following FUS+MB exposure. Expediting the restoration of BBB integrity would be expected to reduce the accumulation of extravasated plasma proteins and to lessen the duration for which the brain is vulnerable to circulating pathogens. It is important to note, however, that depending on the timing of DEX administration, as well as the half-life and size of drug being delivered, extravasation of the therapeutic agent may also be reduced. Given that BBB permeability enhancement decays exponentially with time and in a size-dependent manner following sonication 71, it should be possible to balance treatment efficacy with safety. The administration of DEX following FUS+MB exposure would be most applicable in scenarios for which large molecule therapeutic agents or those with short plasma half-lives are being delivered and a transient inflammatory response is undesirable. In these situations, a majority of the therapeutic agent that will passively extravasate in the targeted location occurs in the several minutes to one hour following sonication 71. In these situations, delayed administration of DEX (E.g. several minutes to one hour following sonication) should reduce the extravasation of bloodborne substances and reduce the magnitude of inflammatory response with only a small reduction in the quantity of therapeutic agent delivered.

Two days following FUS+MB exposure, saline-control animals displayed significantly elevated expression of MCP1 and ICAM1 in sonicated, relative to non-sonicated, dorsal hippocampi. Notably, animals that received DEX administration following exposure did not display these lateralized differences. Previous work has demonstrated the inhibitory effects of DEX on both MCP1 and ICAM1 expression, *in vitro* 72,73 and *in vivo* 36,46. Given the role of these proteins in vascular inflammation and leukocyte endothelial transmigration 74,75, preventing prolonged elevations in their expression may be important for reducing the risk of tissue damage, specifically in the context of repeated FUS+MB exposures in close succession. Further work may be required to determine the dose and administration schedule that can effectively reduce inflammation following repeated FUS+MB exposures while minimizing the adverse effects associated with repeated DEX administration. Conversely, there are several clinical scenarios in which the inclusion of DEX may provide no added benefit, such as in the FUS+MB-mediated delivery of chemotherapeutics to glioblastomas where inflammation may be considered immaterial. This may also be the case for FUS+MB exposures where inflammatory processes could conceivably be involved mechanistically in the outcome, such as amyloid β plaque clearance 49.

While saline-control animals displayed significantly elevated levels of MCP1 and ICAM1 two days following sonication (2.06- and 1.84-fold increase, respectively, relative to non-sonicated dorsal hippocampi), it is important to consider the magnitude of this change in relation to experimental conditions that result in significant, long lasting, tissue damage. Previous work has reported increases in MCP1 and ICAM1 expression of approximately 19- and 5-fold, respectively, relative to contralateral hemisphere, 24 hrs following FUS+MB exposure 19. The expression of ICAM1 was found to be trending upwards at every time point (5 time points) from 0.5 to 24 hrs post-treatment. Of note, the parameters used by Kovacs *et al.* 19 have been shown to result in hemorrhage and persistent tissue damage 25, as well as to produce a significantly greater degree of inflammation than parameters similar to those used in the current study 22. This suggests that while significant increases in the expression of inflammatory markers were detected, the magnitude of this increase did not reach levels previously shown to result in long-term, overt tissue damage.

In addition to the direct measurement of inflammatory markers, GFAP expression was assessed as an indicator of astrocyte activation 76. At 2 and 10 days following FUS+MB exposure, protein expression and immunoreactive density of GFAP, respectively, were significantly elevated in the sonicated dorsal hippocampi of saline-control rats, a result that is consistent with previous reports 32,49. The effect of DEX administration to prevent these changes may be due to its immunosuppressive actions and/or a more rapid restoration of BBB integrity following sonication, leading to reduced accumulation of plasma proteins known to correlate to astrocyte activation 77. Given the enhanced phagocytic role astrocytes play when BBB permeability is increased 78, some degree of activation may be necessary to restore homeostatic conditions. The non-significant trend of increased GFAP immunoreactive density in the sonicated dorsal hippocampus of DEX-treated animals may reflect this process.

Within the CNS, acute inflammation can have a number of downstream effects, some of which have been observed following FUS+MB-mediated BBB permeability enhancement. It may be hypothesized that increases in the expression of VEGF 19,33, along with vascular growth that has been observed post-sonication 33, may be influenced by inflammatory processes. In the current study, DEX administration was shown to prevent an increase in both VEGF expression at 2 days, and the density of small capillaries at 10 days post-FUS+MB exposure. The differential response observed in saline- and DEX-treated animals may be due to the anti-inflammatory effects of DEX, preventing a feedback response that reciprocally links inflammation and VEGF production 79. By preventing an initial spike in the production of proinflammatory mediators, the concurrent and subsequent production of VEGF and vascular growth may be reduced. An important implication of this study is that the biological impacts of FUS+MB exposures in the brain may be altered pharmacologically or by the physiological state of the subject, adding another layer of complexity for predicting treatment outcomes.

Surprisingly, no significant correlations were found between K^trans^, measured at 15 min or 2 hrs post-sonication, and the expression of inflammatory markers or morphological changes, measured at 2- and 10-days post-sonication. This may be explained by a non-linear progression of these processes. For example, collecting samples at a single time point following FUS+MB exposure may capture peaks or valleys in the biphasic expression of specific proteins depending on the initial impact on BBB permeability. Conversely, strong correlations between changes in vascular permeability and the transcription of several inflammatory markers have been observed at 6 hrs following post-FUS+MBs 22. This suggests that the time points of tissue collection in the current study may not have been conducive to the detection of correlations between K^trans^ and the expression of inflammatory markers or morphological changes.

Beyond the effects of DEX, this work also explored the relationship between acoustic emissions and changes in BBB permeability measured by DCE-MRI. This imaging technique allows for a more quantitative measure of vascular permeability than signal intensity changes in contrast-enhanced T1w imaging, a common approach in the field. Others have demonstrated the utility of DCE-MRI in assessing the half-life of increased vascular permeability 59,61, as well as predicting the extravasated concentration of doxorubicin 59,60 and Evans blue 62 following FUS+MB exposure. Imaging parameters and major findings from previously published work involving DCE-MRI and FUS+MB exposures 59-61,80-86 are compared to the present study in **Supplementary Table 1**. These comparisons emphasize the variability in both DCE-MRI methods and magnitude of FUS+MB-mediated BBB permeability enhancement in the field. In the present study, a strong correlation was found between the exposure-average magnitude of 2ƒ emissions and dorsal hippocampal K^trans, 15 min^; no strong correlations were detected with exposure-average or peak magnitude of 0.5ƒ, ƒ, 1.5ƒ, or wideband emissions. McDannold *et al.* previously demonstrated that second and third harmonic emissions strongly correlate to signal intensity changes in contrast enhanced-T1w MRI when employing fixed PNP 87.

The data presented here also suggest that the magnitude of 1.5ƒ emissions during bursts that initiate a software-triggered drop in PNP are not significantly predictive of subsequent BBB permeability enhancement. Additionally, these results suggest that inertial cavitation, as assessed by the presence of wideband emissions, did not contribute substantially to the effects of sonication on vascular permeability. Sun *et al.* previously demonstrated a strong correlation (r^2^ = 0.73) between wideband emissions and mean K^trans^ within the targeted location when employing sonication parameters that induce prolonged (I.e. greater than 48 hrs) BBB permeability enhancement 84. The strong correlation of 2ƒ emissions to K^trans, 15 min^ in the present study emphasizes the notion that while inertial cavitation should be avoided, the modulation of stable cavitation may produce more predictable changes in BBB permeability. This concept has been integrated into closed-loop acoustic feedback control strategies, with promising results 28,30.

### Limitations

One potential limitation of this study is in the use of single-slice DCE-MRI. This approach assumes that changes in vascular permeability measured at the imaging plane are consistent throughout the dorsal hippocampus, as subsequent analyses were performed across this entire brain region. While the geometry of the ultrasound focus is ellipsoidal in the direction of propagation, there may be small variations in BBB permeability above and below the imaging plane. This imaging protocol was designed to achieve adequate signal-to-noise ratio with high temporal and spatial resolution, but at the expense of imaging volume.

Another limitation of this work is the small number of inflammatory markers assessed. Inflammation involves the initiation of a wide range of pathways and changes in the expression, localization, and function of a large number of proteins. Evaluating changes in the expression or immunoreactivity of a limited number of markers at two time points does not capture the complexity of processes that follow FUS+MB exposure. The proteins evaluated in the current study were chosen based on results from previous array-based analyses, implicating their involvement in inflammatory processes following sonication 19,22,32. Further work, however, is required to obtain a more complete picture of the effects of DEX on inflammation following FUS+MB exposure.

### Conclusions

DEX administration following FUS+MB exposure was found to expedite the restoration of BBB integrity in the targeted dorsal hippocampus and to prevent a subsequent elevation in the production of inflammatory markers. These results suggest that DEX may provide a means of modulating the degree to which BBB permeability is increased and may enable repeated FUS+MB exposures with a reduced risk of tissue damage, induced by the accumulation of detrimental effects. Given its widespread clinical use and well documented mechanisms of action, the results presented here suggest that DEX administration following FUS+MB exposure may be warranted in clinical cases in which vascular damage is suspected and the goal of treatment is to restore or preserve neural function.

## Supplementary Material

Supplementary figures and table.

## Abbreviations

AIF: arterial input function
ANCOVA: analysis of covariance
AU: arbitrary units
BBB: blood-brain barrier
CA1-4: cornus amonis 1-4
CD: cluster of differentiation
C_p_: concentration of contrast agent in plasma
C_t_: concentration of contrast agent in tissue
DCE: dynamic contrast-enhanced
DEX: dexamethasone
DG: dentate gyrus
EES: extravascular-extracellular space
ELISA: enzyme-linked immunosorbent assay
f: transmit frequency
FFT: fast Fourier transform
FUS: focused ultrasound
GFAP: glial fibrillary acidic protein
GLUT1: glucose transporter-1
ICAM1: intercellular adhesion molecule-1
IFNɣ: interferon gamma
IL: interleukin
K^trans^: transfer constant
MB: microbubble
MCP1: monocyte chemoattractant protein-1
MRI: magnetic resonance imaging
PB: phosphate buffer
PNP: peak negative pressure
r_1_: longitudinal relaxivity
t: time
T2w: T2-weighted
TE: echo time
TI: inversion time
TIMP1: tissue inhibitor of metalloproteinase-1
TNF: tumour necrosis factor
TR: repetition time
v_e_: fractional EES volume
v_p_: fractional plasma volume
VEGF: vascular endothelial growth factor
