# Supplementary material for: Investigating the effects of dexamethasone on blood-brain barrier permeability and inflammatory response following focused ultrasound and microbubble exposure
Source: Theranostics. 2020 Jan 1;10(4):1604–18. doi: 10.7150/thno.40908 (PMC6993222; doi:10.7150/thno.40908)
Supplement: Supplementary file 1 — Supplementary figures and table. [file thnov10p1604s1.pdf]

Investigating the effects of dexamethasone on blood-brain barrier permeability and inflammatory response following focused ultrasound and microbubble exposure

Dallan McMahon, Wendy Oakden, and Kullervo Hynynen

SUPPLEMENTARY MATERIAL

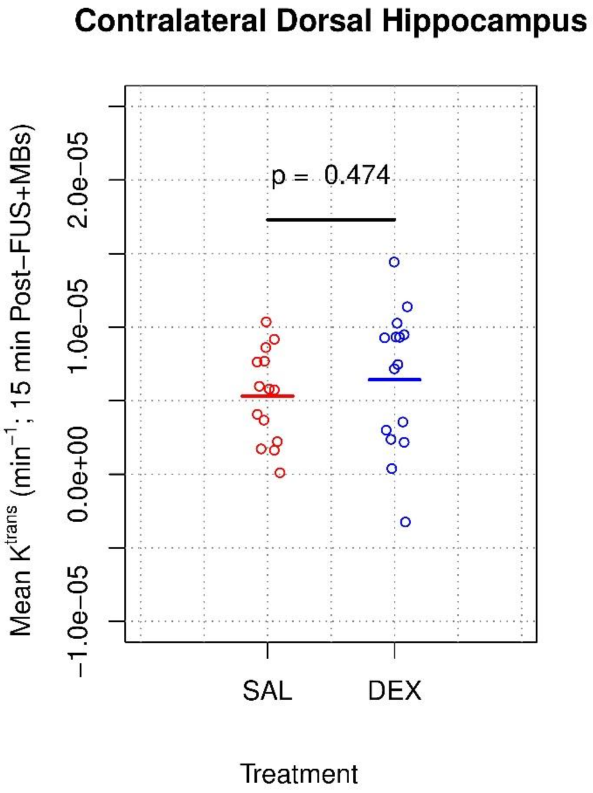

Supplementary Figure 1: Dorsal hippocampal $K^{trans, 15\ min}$ in hemisphere contralateral to sonication. No significant difference was detected in contralateral dorsal hippocampal $K^{trans, 15\ min}$ between groups (saline = 5.31 x 10^{-6} min^{-1} ± 3.17 x 10^{-6} min^{-1}; DEX = 6.42 x 10^{-6} min^{-1} ± 4.81 x 10^{-6} min^{-1}; p = 0.47).

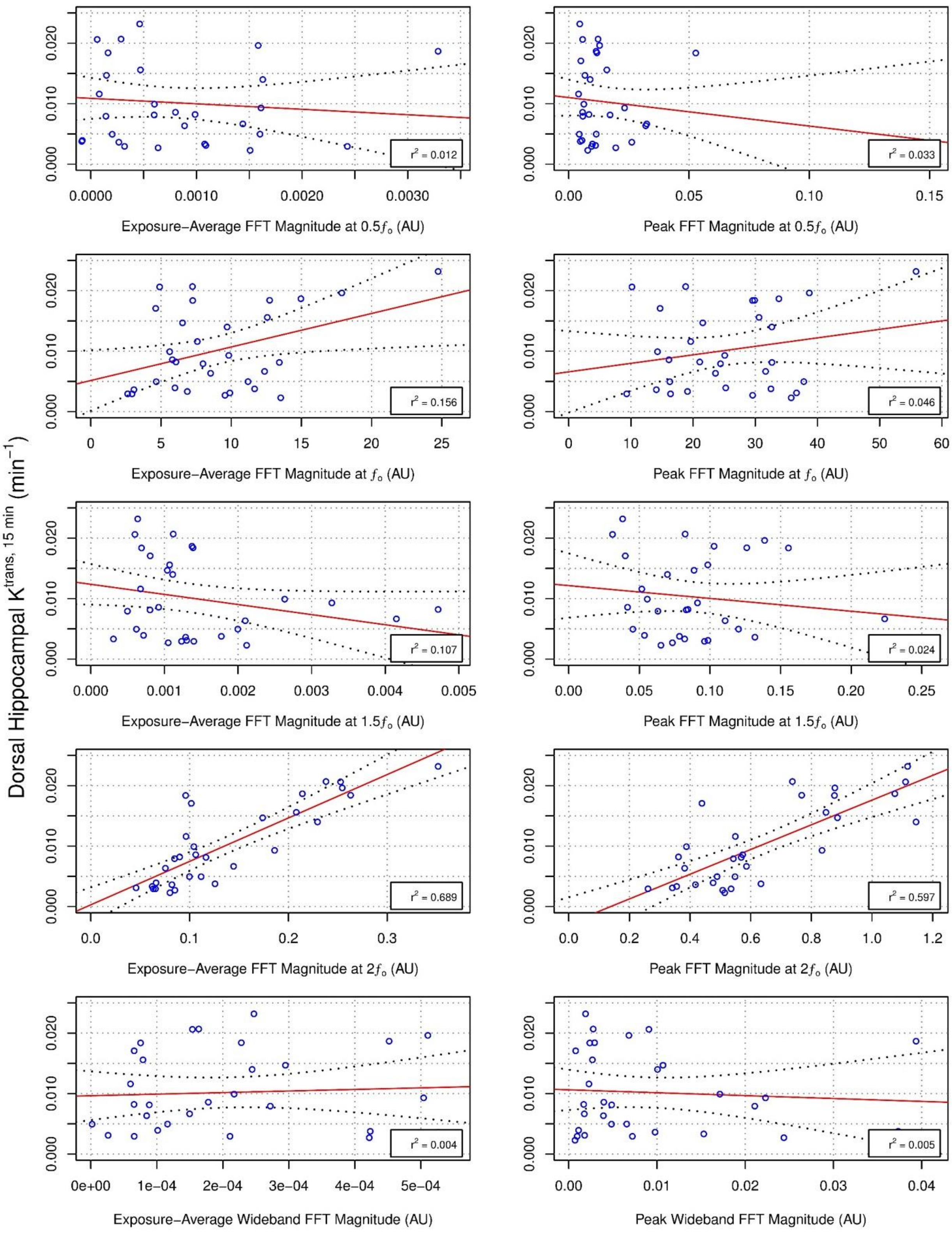

Supplementary Figure 2: Correlation between acoustic emissions and post-FUS+MBs dorsal hippocampal $K^{trans,\ 15\ min}$. Hydrophone signals captured during FUS+MB exposures were analysed retrospectively to explore potential

relationships between $K^{trans, 15 min}$ measurements and spectral characteristics of the acquired acoustic emissions. The exposure-average and peak magnitude of 0.5ƒ, ƒ, 1.5ƒ, 2ƒ, and wideband emissions are displayed in relation to $K^{trans, 15 min}$ in the sonicated dorsal hippocampus. Black dotted lines indicate 95% confidence intervals. AU = arbitrary units.

Supplementary Table 1: Comparison of DCE-MRI methods and findings from select studies investigating FUS+MB exposures in the brain

Reference	Animal Model	Contrast Agent	AIF Method	Main Findings
Vlachos et al. 2010 [80]	C57BL/6 mice	Gadodiamide, Omniscan ® (0.15 mmol/mouse; i.p.)	1. Population average of arterial ROI (internal carotid artery)<br>2. Reference tissue method (temporal muscle)	• Mean K^{trans} calculated to be 0.02 ± 0.0123 min^{-1} and 0.03 ± 0.0167 min^{-1} across targeted tissue volumes using population averaged ROI and reference tissue methods of quantifying AIF, respectively (imaging directly following sonication)<br>• No evidence of tissue damage 7 days post-FUS+MB exposure (H&E)
Vlachos et al. 2011 [81]	C57BL/6 mice	Gadodiamide, Omniscan ® (0.15 mmol/mouse; i.p.)	Population average of arterial ROI (internal carotid artery)	• Mean K^{trans} within targeted volumes ranged from 0.0105 ± 0.0035 min^{-1} (MB diameter = 1-2 µm; mechanical index = 0.37) to 0.0493 ± 0.0063 min^{-1} (MB diameter = 6-8 µm; mechanical index = 0.49)<br>• Higher PNPs and larger MB diameters resulted in greater mean K^{trans} across sonicated tissue volumes (imaging directly following sonication)<br>• Neuronal damage and cell loss evident (H&E) in 7.5% of mice 7 days after sonication at mechanical indexes higher than 0.37 and microbubble diameters larger than 4–5 µm
Park et al. 2012 [59]	Sprague Dawley rats	Gd-DTPA, Magnevist ® (0.125 mmol/kg; i.v.)	Arterial ROI (ophthalmic artery or transverse sinus)	• Mean K^{trans} within targeted volumes were 0.0142 ± 0.006 min^{-1} at 30 min post-FUS+MBs for single sonications and decayed exponentially as a function of time (half-life of 2.22 hrs)<br>• Sonication of the same location twice with delays 10 or 120 min resulted in greater mean K^{trans} and prolonged a half-life of increased BBB permeability<br>• Linear correlation (r^2 = 0.49) between mean K^{trans} at 30 min post-FUS+MB exposure and doxorubicin concentration in sonicated brain tissue ~16 hrs post-FUS+MB exposure

				• Small regions of RBC extravasations (H&E) evident in a subset of animals 4 hrs following FUS+MB exposure
Yang et al. 2014 [82]	Fischer 344 rats (F98 glioma)	Gadodiamide, Omniscan ® (1 mmol/kg)	Arterial ROI (cerebral artery)	• Mean K^{trans} within targeted tumours were 0.128 ± 0.019 min^{-1} at 20 min and 0.103 ± 0.023 min^{-1} at 24 hrs following sonication (higher than non-sonicated tumours by 2.46-fold at 20 min and 1.78-fold at 24 hrs)<br>• Mean K^{trans} within targeted tumours at 20 min post-FUS+MB exposure correlated (r^2 = 0.9) to Evans blue dye concentration 4 hrs following administration<br>• Small regions of RBC extravasations (H&E) evident in 2 of 2 animals 20 min and 4 hrs following FUS+MB exposure
Chai et al. 2014 [61]	Sprague Dawley rats	Gd-DTPA, Magnevist ® (0.15 mmol/kg; i.v.)	Venous ROI (venous sinus)	• Mean K^{trans} within targeted volumes were 0.0086 ± 0.0009 min^{-1} and 0.63 and 0.0131 ± 0.0015 min^{-1} at ~10 min post-FUS+MB exposure with mechanical indexes of 0.63 and 1.26, respectively.<br>• Approximately 31% and 42% reduction in mean K^{trans} between ~10 min and 2 hrs post-FUS+MB exposure with mechanical indexes of 0.63 and 1.26, respectively<br>• Half-life of increased BBB permeability estimated to be 2.09 and 5.39 hrs with mechanical indexes of 0.63 and 1.26, respectively<br>• Linear correlation between mean K^{trans} at ~10 min post-FUS+MB exposure and Evans blue dye concentration in sonicated brain tissue 4 hrs post-FUS+MB exposure (r^2 = 0.83 and 0.74 with mechanical indexes of 0.63 and 1.26, respectively)<br>• Regions of RBC extravasations (H&E) evident 6-24 hrs following FUS+MB exposures with mechanical index of 1.26
Aryal et al. 2015 [83]	Sprague Dawley rats (9L gliosarcoma)	Gd-DTPA, Magnevist ® (0.25 mmol/kg; i.v.)	Arterial ROI (ophthalmic artery or transverse sinus)	• FUS+MB exposure increased mean K^{trans} from 0.0077 ± 0.0053 min^{-1} to 0.0129 ± 0.0064 min^{-1} within tumours 9 days after implantation (imaging immediately following sonication)<br>• Mean K^{trans} within tumours were not significantly increased when sonicated 14 or 17 days after implantation<br>• Doxorubicin concentration was increased in sonicated tumours for all implantation time points despite no

				detectable increase in mean K^{trans} at 14 or 17 days after implantation<br>• Linear correlation (r^2 = 0.44) between mean K^{trans} immediately following sonication and doxorubicin concentration at 2 hrs post-FUS+MB exposure (9 days following tumour implantation)<br>• Small regions of RBC extravasations (H&E) evident 4 hrs following FUS+MB exposure in two animals assessed
Sun et al. 2015 [84]	C57BL/6 mice	Gadodiamide, Omniscan ® (0.15 mmol/mouse; i.p.)	Population average of arterial ROI (internal carotid artery)	• With MBs 1-2 µm in diameter and mechanical index of 0.24, mean K^{trans} across the targeted volume was 0.004 ± 0.010 min^{-1} (immediately following sonication) and remained elevated for 0.2 ± 0.4 days<br>• With MBs 4-5 µm or 6-8 µm in diameter, all FUS+MB exposure parameters explored induced BBB permeability enhancement that persisted for at least 48 hrs (up to 5 days)<br>• Magnitude of both wideband emissions (r^2 = 0.73) and harmonic + ultraharmonic emissions (r^2 = 0.82) correlated to mean K^{trans} assessed immediately following FUS+MB exposure<br>• RBC extravasation and dark neurons (H&E) were evident 7 days following sonication in animals for which BBB permeability enhancement persisted past 48 hrs post-FUS+MB exposure
Chu et al. 2016 [85]	Sprague Dawley rats	Gd-DTPA, Magnevist ® (0.15 mmol/kg; i.v.)	Venous ROI (venous sinus)	• Mean K^{trans} across the sonicated tissue volumes ranged from 0.0061 min^{-1} to 0.0136 min^{-1} for FUS+MBs exposures with mechanical indexes of 0.41 to 1.12, respectively (imaging immediately following sonication)<br>• Linear correlation (r^2 = 0.97) between mechanical index of FUS+MB exposure and mean K^{trans} across the sonicated volume for transmit frequencies of 0.4 or 1 MHz
Park et al. 2017 [60]	Sprague Dawley rats (9L gliosarcoma)	Gd-DTPA, Magnevist ® (0.25 mmol/kg; i.v.)	Arterial ROI (ophthalmic artery or transverse sinus)	• FUS+MB exposure increased mean K^{trans} from 0.016 ± 0.0069 min^{-1} to 0.032 ± 0.0085 min^{-1} within tumours (imaging immediately following sonication)<br>• Mean K^{trans} across the sonicated volume of non-tumour tissue was 0.019 ± 0.0054 min^{-1} (imaging immediately following sonication)<br>• Linear correlation (r^2 = 0.56) between mean K^{trans} across either tumour or non-

				tumour tissue immediately following sonication and doxorubicin concentration at 1 or 24 hrs post-FUS+MB exposure<br>• Small regions of RBC extravasations (H&E) evident 4 hrs following FUS+MB exposure in two animals assessed
Samiotaki et al. 2017 [86]	Rhesus macaques	Gadodiamide, Omniscan ® (0.1 mmol/kg; i.v.)	Arterial ROI (not specified)	• Mean K^{trans} across the sonicated tissue volumes ranged from ~0.0001 min^{-1} to ~0.0003 min^{-1} for FUS+MBs exposures with mechanical indexes between 0.28 and 0.57 (imaging ~20 min following sonication)
Present study	Sprague Dawley rats	Gadobutrol, Gadovist ® (0.4 mmol/kg; i.v.)	Reference tissue method (temporal muscle)	• Mean K^{trans} across the sonicated tissue volumes 15 min following FUS+MB exposure was 0.013 ± 0.0053 min^{-1}<br>• Reductions in mean K^{trans} between 15 min and 2 hrs post-FUS+MB exposure of 60.8% ± 9.7% and 74.2% ± 10.4% in animals that received saline and DEX, respectively<br>• Linear correlation (r^2 = 0.689) between exposure-averaged second harmonic emissions and mean K^{trans} across the sonicated tissue volumes 15 min following FUS+MB exposure
